# Variation in body condition, corticosterone response and immune function is related to the timing of nesting in Franklin’s Gull

**DOI:** 10.1093/conphys/coaf024

**Published:** 2025-04-11

**Authors:** Shawn Weissenfluh, Jeffrey Kittilson, Penelope Gibbs, Wendy L Reed, Mark E Clark

**Affiliations:** Department of Biological Sciences, North Dakota State University, 1340 Bolley Drive, 201 Stevens Hall, Fargo, ND 58108-6050, USA; Department of Biological Sciences, North Dakota State University, 1340 Bolley Drive, 201 Stevens Hall, Fargo, ND 58108-6050, USA; Department of Microbiological Sciences, North Dakota State University, 1230 Albrecht Blvd, Fargo, ND 58108-6050, USA; Department of Biology, University of Minnesota Duluth, 1035 Kirby Drive, Duluth, MN 55812, USA; Department of Biology, University of Minnesota Duluth, 1035 Kirby Drive, Duluth, MN 55812, USA

**Keywords:** Condition, corticosterone response, migratory bird, timing of breeding

## Abstract

Understanding individual variation in adult condition is necessary for developing hypotheses on how nest initiation, chick development and recruitment are related in many migratory birds. We quantified attributes of condition among Franklin’s Gull (*Lecuophaeus pipixcan*) adults initiating nesting at different dates during the nesting period using four metrics: body measurements recorded from live-trapped birds, the corticosterone levels measured from blood samples collected serially from live-trapped birds, heterophil/lymphocyte ratios determined from blood smears and antimicrobial capacity of plasma. Variation in physiological condition was related to the timing of nesting such that individuals nesting later in the season had lower mass relative to skeletal size, increasing corticosterone concentrations measured 3-, 20- and 30-minute post-capture and reduced immune performance. Specifically, residual body mass decreased and keel bone exposure increased with laying date. Additionally, birds nesting later in the season show higher maximum corticosterone concentrations after exposure to acute capture stress along with reduced bacteria-killing capability of their plasma. Our findings indicate that timing of nesting is significantly related to the physiological condition of Franklin’s Gull. Individual variation in condition may be related to time constraints observed in temperate latitudes and whether these birds are capital (i.e. acquiring resources outside the breeding area) or income (i.e. acquiring resources locally) breeders. Quantifying variation in physiological condition within the breeding season will aid in modelling population-level response to shifts in nesting phenology.

## Introduction

As climate change affects breeding phenology of migratory birds, there is a knowledge gap in how timing of nesting affects adult survival and future reproduction. Shifts in the date of arrival on the breeding grounds have been documented in many species (Newson *et al.*, 2016; Ward *et al.*, 2016). The timing of arrival, and subsequently nesting, likely constrain recruitment because the energy demands of developing chicks must match periods of food abundance, predator activity, weather or other environmental factors (Durant *et al.*, 2007; Dunn *et al.*, 2011). In birds, timing of nesting and offspring development can be related (Catry *et al.*, 1998; Arnold *et al.*, 2004; Greño *et al.*, 2008; Hipfner *et al.*, 2010; Hirose *et al.*, 2012; DiMatteo and Clark, 2017), and offspring growth rate, age at independence and sex can affect juvenile survival (Maness and Anderson, 2013). However, understanding how the timing of nesting is related to adult fitness and future reproduction has received less attention.

Environmental cues that affect arrival date and nest initiation can alter physiology, flight muscle and condition of breeding adults. For example, in the northern hemisphere photoperiods change such that day-length increases as the Julian day approaches the summer solstice, which initiates an array of physiological changes in birds including gonad recrudescence, feather moult, fat deposition and flight muscle (Dietz *et al.*, 1999; Wingfield, 2005; Wikelski *et al.*, 2008; Price *et al.*, 2011). Temperature can also affect physiology and the timing of breeding. Trumpeter Finch (*Bucanetes githagineus*) females delay egg-laying dates up to 40 days due to lower environmental temperatures (Barrientos *et al.*, 2007), and laying date advances with increasing environmental temperatures in Great Tit (*Parus major*) (Caro *et al.*, 2013). Intercontinental migratory birds experience an especially short amount of time to nest, fledge their young and prepare the offspring (and themselves) for the migration back to their wintering grounds. Thus, the constraints on timing of nesting may manifest in multiple attributes of condition or physiological state. Metrics for overall body condition, glucocorticoid profile and immune function have been developed for, and applied to, several avian species.

Adult condition affects both current and future reproductive success. Body condition of Great Tit females is positively correlated with reproductive output (Norte *et al.*, 2010). However, studies have also shown that condition is positively correlated with nutritional reserves critical for adult survival (Brown, 1996) and therefore future offspring production. It is hypothesized that changes in adult condition across the breeding season are a manifestation of trade-offs between investments to offspring versus self-maintenance predicated on nest initiation date, because offspring survival to recruitment generally declines across the nesting season (Drent, 2006) [and reviewed in Maness and Anderson (2013)].

Condition of nesting adults can be quantified through several measures, including body measurements recorded from live-trapped birds. Body mass often fluctuates within the breeding season. Kittiwake (*Rissa tridactyla*) females exhibit an increase in body mass during incubation and a decrease in body mass during the chick-rearing period (Moe *et al.*, 2002). Similarly, Arctic Skua (*Stercorarius parasiticus*) females retain body fat reserves during incubation but shed those reserves after nesting, which reduces wing loading (the ratio of wingspan to body mass) and improves flight efficiency (Phillips and Furness, 1997). Quantifying (e.g. ratio, residuals in regression) the relationship between body mass and tarsus or wing length can be an indicator of whole-body condition, fat storage or lean mass (Richner *et al.*, 1993; Gosler *et al.*, 1998; Green, 2001). Keel depth, a measure of the protrusion of the keel bone above breast muscle tissue, is another measure of whole organism size but more specific to flight proficiency that is also known to vary in migratory birds during breeding (Hatch *et al.*, 2010). Similarly, wing loading represents a body condition metric specific to flight capability (Mueller *et al.*, 2002). Residuals of body mass regressions with skeletal size (e.g. tarsus length) are assumed to quantify body condition (Green, 2001; Schulte-Hostedde *et al.*, 2005). However, keel depth and wing loading are expected to be negatively correlated with body condition and flight muscle performance (Gosler *et al.*, 1998; Schmidt-Wellenburg *et al.*, 2008; Hatch *et al.*, 2010).

The corticosterone response in birds provides a measure of an individual’s physiological response to an acute stressor. Perceived threats or short-term stressors illicit an increase in the production (and circulating levels) of corticosterone that precipitates the vertebrate fight or flight response. As such, capture and handling of free-living birds can induce a rapid elevation of plasma corticosterone concentrations above the background baseline concentration. Because repeated exposure to stress may alter the corticosterone response (Cyr and Romero, 2007), integrating the corticosterone concentration versus time curve has been used to quantify glucocorticoid-mediated changes to physiological condition (Breuner and Hahn, 2003). The corticosterone response is repeatable, perhaps because it is linked to personality (Grace and Anderson, 2014) or shaped by early life interactions (Grace and Anderson, 2018; Grace *et al.*, 2020), and therefore provides a robust measure of long-term physiological effects of exposure to an acute stress. The corticosterone response has been quantified by the difference between maximum and minimum concentrations measured over time, which is expected to be positively correlated with short-term stress exposure (Cyr and Romero, 2007). Indeed, magnitude of the corticosterone response was negatively correlated with parental effort in Mourning Doves (*Zenaida macroura*) (Miller *et al.*, 2009).

Chronic or long-term effects of stress in birds have been measured from changes in the relative numbers of immune cells in the blood. Specifically, the heterophil/lymphocyte ratio has been used to quantify long-term physiological effects. Long-term social conditions (i.e. larger group size), nesting and injuries were linked to individuals exhibiting increases in the heterophil/lymphocyte ratio (Gross and Siegel, 1983; Vleck *et al.*, 2000). Greater investment in offspring was correlated with higher heterophil/lymphocyte ratio in adult Great Tits (Ots and Hõrak, 1996) and was negatively related to long-term survival in Nazca Boobies (*Sula granti*) (Maness *et al.*, 2023).

Physiological changes occurring with the timing of nesting may correlate with the immune function. Infection delays migration to breeding areas (Risely *et al.*, 2018), and immune-challenged Dark-eyed Juncos *Junco hymelis* delayed egg laying (Needham *et al.*, 2017). In free-living birds, antimicrobial capacity of plasma has been used to characterize the status of the immune system (Liebl and Martin, 2009). Bactericidal capacity of plasma assesses multiple components of constitutive immunity in vertebrates, and has been correlated with handling stress in birds (Millet *et al.*, 2007). Bacteria-killing capacity of nesting adult Zebra Finches (*Taeniopygia guttata*) decreased from incubation to nestling period (Evans *et al.*, 2016) and was positively correlated with the colour of female Spotless Starlings (*Sturnus unicolor*) prior to nesting (Ruiz-Rodríguez *et al.*, 2020). In tropical birds, exposure to 1 hour of acute stress was shown to reduce bacteria-killing ability by up to 40% (Matson *et al.*, 2006). Hence, the estimated numbers of bacteria in a blood plasma culture (after an interval of time for bacterial growth) are expected to be negatively correlated with immune function (Millet *et al.*, 2007).

Effects of timing of nesting have been observed in Franklin’s Gull (*Leucophaeus pipixcan*), an intercontinental migrant that nests in the northern Great Plains. Berg (2009) found that Franklin’s Gull chicks hatched early in the season were structurally larger than chicks hatched later in the season. Franklin’s Gull eggs artificially incubated under photoperiods representative of early and late seasons indicated that both photoperiod and maternal effects associated with timing of nesting affect embryonic development of chicks (Clark and Reed, 2012). Franklin’s Gull eggs laid later in the season contain higher concentrations of maternally derived testosterone and thyroid hormone (Boertje *et al.*, 2019), both of which can increase the growth rates (Eising *et al.*, 2001; Ruuskanen *et al.*, 2016). Timing of nesting also affects post-hatching growth, as eggs laid later in the season produce chicks that achieve faster growth in mass and wing feathers than chicks from eggs laid early in the season (Reed and Clark, 2016). However, it is not known if adult condition or physiology varies with the timing of nesting in Franklin’s Gull. Changes in adult physiology could be responsible for the seasonal maternal effects observed in Franklin’s Gull embryo and chick development.

Franklin’s Gull is a species of concern in the Northern Great Plains of North America (Hagen *et al.*, 2005; Minnesota Department of Natural Resources (MN DNR), 2006; Idaho Department of Fish and Game, 2009; Montana Natural Heritage Program, Montana Fish Wildlife and Parks, 2009; Oregon Department of Fish and Wildlife, 2016), and agencies responsible for management of this species need information on how the timing of nesting impacts current and future reproduction if changes in climate or length of the breeding season occur. We examined metrics from multiple categories (body size metrics, corticosterone response, heterophil/lymphocyte ratio and immune function) of individual state in adults initiating nesting at different dates to develop hypotheses on how timing of nesting and physiology are related in Franklin’s Gull. Furthermore, we offer our approach as a template for evaluating the effects of the timing of reproduction in other migratory species to develop conservation plans in response to shifts in the phenology of breeding associated with climate change.

## Materials and Methods

### Ethics statement

All work was conducted in accordance with permits from North Dakota Game & Fish Department and the US Fish & Wildlife Service. All work was conducted in accordance with North Dakota State University Institutional Animal Care and Use Committee (IACUC protocol A10067).

### Study site

We monitored adult Franklin’s Gulls during nesting from early May through late June in 2010 at Rush Lake Waterfowl Production Area (48.377972° N, 100.218095° W) in north–central North Dakota. In early May, we observed numerous adult Franklin’s Gulls near the Rush Lake Waterfowl Production Area and began searching areas within the Rush Lake marsh each week to locate nests for monitoring. When a new nest was found, we marked the location with a handheld GPS, placed a small float with a unique nest identification code near the nest, recorded the number of eggs present, marked the eggs on the blunt end with a permanent marker and returned to the nest on subsequent days to determine the final clutch size and the onset of continuous incubation by the adults. Newly initiated nests (i.e. those for which the female was still in the process of laying eggs) were determined by the presence of a single egg in the nest or confirmation of laying by the appearance of another egg within 48 hours. For nests found with one egg present, we used the flotation method to determine if it was recently laid. Eggs that did not float in the water were considered recently laid (Nol and Blokpoel, 1983; Ackerman and Eagles-Smith, 2010). Nests determined to be within the first days of continuous incubation (or not yet undergoing continuous incubation) were targeted for subsequent capture of one adult within the first week of incubation to measure physiological condition. We assumed all nests represented the first attempt (for the season) at breeding for the adults because Franklin’s gull initiates nests for approximately four weeks and is not known to renest (Burger, 1974; Burger and Gochfeld, 2009; Clark and Reed, 2012).

### Adult capture, blood and DNA collection

We quantified physiological condition from adults captured within the first week of continuous incubation that initiated nests early, middle and late in the nesting period observed in the population. Adults were captured using nest traps placed on targeted nests (Burger, 1971). Larger traps were used to acclimate a nesting bird, by allowing them to fly in and out of the trap without disturbance for 1–2 days prior to capture. Smaller traps, which did not allow birds to fly out, were then set to capture the nesting bird. These traps were monitored for captures at 30–45-minute intervals. When inside a trap (both large and small versions), birds assumed normal incubation behaviour. Once an adult was captured (i.e. inside a small trap), we recorded the time of day, approached the nest rapidly and recorded the start time of a startle response, which we assumed began when the individual stood up from incubation or gave an alarm call. The startle response usually began as soon as a bird made visual contact with us as we approached the nest. Upon capture, we collected a blood sample (approximately 600 μl) from the brachial vein, recorded time elapsed from the startle response (with a target of within three minutes), then collected subsequent blood samples (approximately 300 μl each) at 20 minutes and 30 minutes after the time of startle response, which is the protocol for obtaining samples to profile the plasma corticosterone response (Wingfield *et al.*, 1982). Following collection, blood samples were temporarily placed in a cooler of ice for transport to the laboratory. In the interval between collection of the first and second blood samples, we measured body mass using a spring scale (±5.0 grams), tarsus length and keel depth (i.e. protrusion of the sternum above the pectoral muscle at a standard location on the breast) using digital callipers (±0.1 mm), and wing chord length using a wing rule (±0.5 mm). During blood sample collection, we made a blood smear for counting heterophil and lymphocyte numbers, prepared by spreading a drop of blood across a microscope slide producing a single layer of cells. We also set aside a drop of blood in Queen’s lysis buffer (Seutin *et al.*, 1991) to preserve DNA for sexing because Franklin’s Gull is not sexually dimorphic in size or plumage (Burger and Gochfeld, 2009).

Blood samples were taken to the laboratory for processing within approximately 4 hours of collection. We centrifuged blood samples at 1700 G for 10 minutes to separate red blood cells from plasma, and plasma samples were separated into approximately 100 μl of aliquots and stored at −20°C until further analysis (corticosterone radioimmunoassay or bactericidal assay). DNA from red blood cells in lysis buffer was extracted using a Qiagen DNeasy kit (Qiagen, Inc.), following the manufacturer’s protocol, and amplified by PCR with primers 2550F and 2718R, following methods outlined by Fridolfsson and Ellegren (1999).

### Body size metrics

We used three metrics (body mass relative to tarsus length, keel depth and wing chord) of size to infer aspects of condition relative to flight. We computed the residual of body mass from an orthogonal regression of body mass and tarsus length to quantify mass relative to skeletal size (Schulte-Hostedde *et al.*, 2005), but see Peig and Green (2009) for a discussion of alternatives to scaling mass to skeletal size. Keel depth was used to quantify flight muscle size (Bolton *et al.*, 1991; Schmidt-Wellenburg *et al.*, 2008). Finally, we used the wing chord length to quantify skeletal size specific to lift (Mueller *et al.*, 2002). We report pairwise correlations between the metrics to justify our interpretation of condition with respect to flight ability.

### Heterophil/lymphocyte counts

We also quantified potentially long-term variation in relative leukocyte abundance among individuals using heterophil/lymphocyte counts. We counted leukocytes (i.e. heterophils, lymphocytes, basophils, eosinophils and monocytes) from prepared blood smears as described in Clark *et al.* (2009). Blood smears were fixed and stained with a Harleco® Hemacolor® staining kit. After staining, leukocytes were manually counted from smears observed at 100x magnification (oil immersion) on a compound microscope. At least 100 cells (heterophils, lymphocytes, basophils, eosinophils and monocytes combined) per smear were counted to provide the ratio between heterophils and lymphocytes (Vleck *et al.*, 2000).

### Radioimmunoassay

We determined plasma corticosterone concentrations using radioimmunoassay to quantify the corticosterone response in nesting adults. We used 10 μl of plasma for the corticosterone radioimmunoassay following the protocol described by Wingfield and Farner (1975). Briefly, a small amount of ^3^H corticosterone (2000 cpm) was added to plasma samples to estimate the extraction efficiency. We extracted steroids from the plasma by adding 5 ml of distilled dichloromethane and dried the supernatant at 40°C under a stream of nitrogen gas. Dried extracts were re-suspended in PBSg buffer and refrigerated overnight at 4°C. We split these samples into duplicate vials for the radioimmunoassay and estimated corticosterone levels based on competitive binding between known amounts of labelled corticosterone and unknown amounts of corticosterone in samples on a corticosterone-specific antibody (cross-reactivity binding affinity for corticosterone antibody ab7798: 11-dehydrocorticosterone 0.67%, deoxycorticosterone 1.5%, 18-OH-DOC <0.01%, cortisone <0.01%, cortisol <0.01% and aldosterone 0.2%). In each radioimmunoassay, a standard curve and two-standard samples of known concentrations were included to estimate within and among assay variation.

### Bactericidal assay

Immune system strength was quantified using a bactericidal assay. Antimicrobial activity was measured following the protocol described by Millet *et al.* (2007). Briefly, we cultured our complement-sensitive (i.e. positive control) isolate (A1, *E. coli* Serotype O32 obtained from healthy chickens *Gallus gallus*) and complement-resistant (i.e. negative control) isolate (V1, *E. coli* non-typable obtained from the liver of a domestic chicken with systemic *E. coli* infection) controls on tryptic soy agar (TSA) and incubated overnight at 37°C. After incubation, we inoculated 3.0 ml of tryptic soy broth (TSB) with a single colony from each of the controls and incubated overnight at 37°C, while shaking at 200 rpm. Following overnight incubation, 10 μl of each of the controls were used to inoculate 3.0 ml of TSB, which were then incubated at 37°C while shaking at 200 rpm for 45 minutes. Next, two 100 μl of aliquots of each sample were centrifuged at 3600 G at 4°C for 5 minutes. We then decanted the supernatant and re-suspended each sample with 100 μl of PBS, vortexed each sample, and then repeated centrifugation. The samples were again decanted and re-suspended with 60 μl of PBS and 20 μl of plasma sample, which were incubated at 37°C. After vortexing, 5 μl of each sample was removed and placed into a separate vial containing 45 μl of PBS. Serial dilutions (1:10) were then performed for each sample and diluted to 10^−7^ and plated on TSA in 3 × 10 μl aliquots for each dilution. The subsample and serial dilution step occurred at 0-, 2- and 4 hours during incubation. After each sample was plated, the plates were incubated at 37°C overnight. After overnight incubation, we counted and recorded the number of colonies on the plates, and then estimated the number of bacteria in the culture (at the 0-, 2- and 4-hour period) based on the dilution.

### Statistical analysis

We used general linear models to characterize condition, corticosterone response, heterophil/lymphocyte ratios and immune function of Franklin’s Gull adults initiating nesting at different dates. Specifically, we modelled heterophil/lymphocyte ratio and bactericidal efficacy of plasma (quantified by A1 and V1 colony counts at 2- and 4-hour incubation) each with a linear model including the fixed effects of sex, trap day (expressed as day of the year) and the interaction between sex and trap day. We modelled the size metrics (i.e. residuals of body mass-tarsus, keel depth and wing chord) with a linear model including the fixed effects of sex, trap day (expressed as day of the year), the interaction between sex and trap day, and time of day (expressed as hours past midnight). We used trap day rather than nest initiation date because: (i) trap day was known (i.e. measured without error) for all nests, whereas initiation date was estimated by flotation method for some nests (i.e. measured with error), (ii) trap day was correlated (with ρ > 0.94, *P* < 0.001, *n* = 30) with initiation date for nests with known initiation date, and (iii) all nests were trapped within the first 10 days of incubation. We then constructed a suite of sub-models by eliminating a single factor from the full model (e.g. sex + trap day + sex × trap day) beginning with the interaction term (or time of day in the size metrics models), and proceeded until all factors in the model were removed, computed the relative Akaike Information Criteria adjusted for small size (ΔAIC_c_) (Akaike, 1973; Burnham and Anderson, 2002) and log-likelihood for each model in the suite, and identified uninformative parameters based on the algorithm developed by Leroux (2019) to select a final model without uninformative parameters. In final models for which the parameter for the sex term was excluded (i.e. uninformative), we evaluated this *post hoc* model by including observations from individuals for which we were unable to determine sex using DNA (and therefore, sample size increased in the *post hoc* model). To model corticosterone concentrations, we used a mixed model with repeated measures with effects of sex, trap day, interaction between sex and trap day, serial sample number (first, second or third), time of day (expressed as hours past midnight) and adult ID as a random effect. We compared successive plasma corticosterone concentrations with a Tukey test of the least-square means from the repeated measures model and verified that the baseline (i.e. first) concentration was not correlated with the time elapsed from the startle response. We then constructed a suite of sub-models by eliminating a single fixed factor from the full-repeated-measures model (i.e. sex + trap day + sex × trap day + serial sample number + time of day + adult ID) beginning with the time of day term, and proceeded until all factors in the model were removed as described above, computed the ΔAIC_c_ and log-likelihood for each model in the suite, and identified uninformative parameters following Leroux (2019) to select a final model without uninformative parameters. In final models for which the parameter for the sex term was excluded (i.e. uninformative), we evaluated this *post hoc* model by including observations from individuals for which we were unable to determine sex using DNA (and therefore, sample size increased in the *post hoc* model). We log-transformed heterophil/lymphocyte ratios and the A1 and V1 bacterial colony counts to achieve normality, but all other dependent variables were normally distributed. Finally, we checked pairwise correlations between the residual body mass, keel depth and wing chord, and for correlations between heterophil/lymphocyte ratio and the A1 and V1 bacterial colony (log-transformed) counts at 2 and 4 hours. All statistical analyses were conducted using JMP statistical software (SAS Institute, 2023).

## Results

We captured 61 adults from 61 nests determined to be in the first week of continuous incubation from 14 May 2010 to 16 June 2010 at the Rush Lake Waterfowl Production Area in North Dakota. Of the 61 adults captured, we obtained body mass measurements from 54 individuals, tarsus length measurements from 55 individuals, keel depth measurements from 49 individuals and wing chord from 49 individuals. We were able to obtain at least one plasma sample from all 61 captured adults but measured plasma corticosterone concentrations from serially collected samples (i.e. concentrations from plasma samples collected at approximately 3, 20 and 30 minutes from startle response) from 55 of the individuals. The initial plasma sample was collected in less than 4 minutes (mean and standard error of 3.10 ± 0.16 minutes) for all but four individuals (three of which were collected in less than 5 minutes, and one in 5.33 minutes). When we excluded these four individuals from the analysis of corticosterone response, results of the analysis did not change, so we report the results of all 55 individuals. The second and third plasma samples were collected at a mean of 19.89 ± 0.13 and 29.41 ± 0.21 minutes, respectively. We obtained blood smears to determine heterophil/lymphocyte ratio for 54 individuals and recovered plasma and cultured the A1 and V1 bacteria assays from 56 individuals. We successfully extracted and amplified DNA for sex determination of 45 individuals (24 of which were females and 21 of which were males), and we had collected three serial plasma samples of 41 of these (23 of the females and 18 of the males).

**Figure 1:**
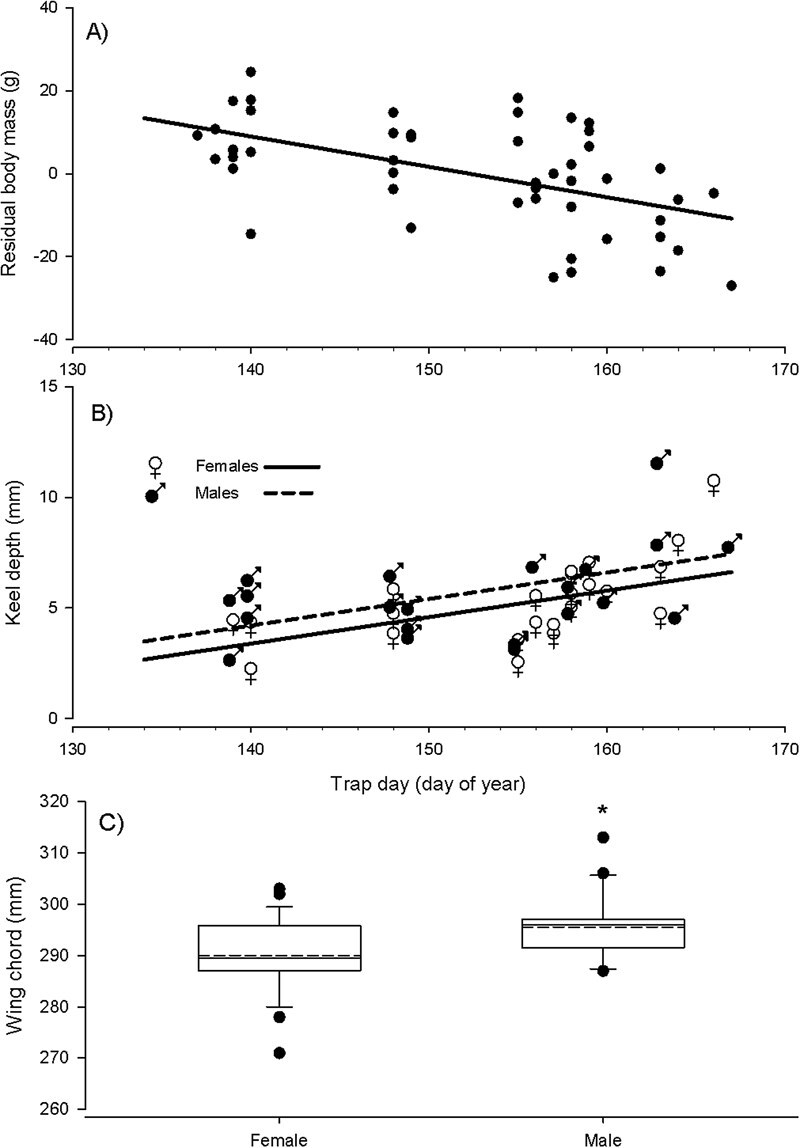
For Franklin’s Gull adults captured in the first half of incubation, variation in (**A**) residual body mass (from the orthogonal regression of body mass and tarsus length) was best explained by trap day, (**B**) keel depth (i.e. exposure of the keel bone above the pectoral muscle) by sex and trap day (expressed as ordinal day of the year), but (**C**) wing chord only differed between females and males. Filled circles indicate observed values, except in (B) where ♀ and ♂ indicate observed values for females and males, respectively. The solid line in (A) represents the linear regression fit to the residual body mass (*F*_1,47_ = 18.42, *P* < 0.001, *r*^2^ = 0.28) and the lines in (B) represent the regression for females (solid line) or males (dashed line) for the trap day + sex effect model of keel depth (*F*_2,41_ = 8.21, *P* = 0.004, *r*^2^ = 0.29). In (C) boxes indicate 25th through 75th percentiles with 5th and 95th percentile caps (and outlier points), solid lines for medians, dashed lines for means and asterisk indicating significant differences in means (*F*_1,43_ = 7.79, *P* = 0.008, *r*^2^ = 0.15)

Residual body mass and keel depth were related to trap day, wing chord was not related to trap day, and none of the size metrics were related to time of day. Adult body mass and right tarsus length were positively correlated (ρ = 0.45, *P* = 0.001, *n* = 49), and we determined residual body mass from an orthogonal regression fit to the observations (body mass = 15.03⋅and tarsus length—370.16). Trap day, sex, the interaction between trap day and sex, and time of day (at capture) explained over 24% of the variation in residual mass (*F*_4,39_ = 3.23, *P* = 0.022, *r*^2^ = 0.25, ΔAIC_c_ = 6.75), but only trap day had a significant (negative) effect (*F*_1,39_ = 12.38, *P* = 0.001, *r*^2^ = 0.24) (Table 1). Comparisons with a model including trap day, sex and the interaction between trap day and sex (*F*_3,40_ = 4.40, *P* = 0.009, *r*^2^ = 0.25, ΔAIC_c_ = 4.11), a model including trap day and sex (*F*_2,41_ = 6.32, *P* = 0.004, *r*^2^ = 0.24, ΔAIC_c_ = 2.27) and a model (which was most parsimonious) including only trap day (*F*_1,42_ = 12.77, *P* = 0.001, *r*^2^ = 0.23, ΔAIC_c_ = 0) indicated only the trap-day parameter was informative (Table 1). A *post hoc* model with only trap day (and *n* = 49) also indicated residual body mass was lower for later trap day (*F*_1,47_ = 18.43, *P* < 0.001, *r*^2^ = 0.28; Fig. 1A, Table 1). Similarly, trap day, sex, the interaction between trap day and sex, and time of day explained 32% of the variation in keel depth (*F*_4,39_ = 4.64, *P* = 0.004, *r*^2^ = 0.32, ΔAIC_c_ = 2.95), but only trap day had a significant (positive) effect (*F*_1,39_ = 13.88, *P* < 0.001, *r*^2^ = 0.24) (Table 1). Comparisons with a model including trap day, sex and the interaction between trap day and sex (*F*_3,40_ = 5.66, *P* = 0.003, *r*^2^ = 0.30, ΔAIC_c_ = 1.81), a model (which was most parsimonious) including trap day and sex (*F*_2,41_ = 8.21, *P* = 0.001, *r*^2^ = 0.29, ΔAIC_c_ = 0; Fig. 1B), and a model including only trap day (*F*_1,42_ = 13.30, *P* < 0.001, *r*^2^ = 0.24, ΔAIC_c_ = 0.29) indicated the parameters for the interaction term and time of day were uninformative (Table 1). Variation in wing chord was not explained by a model with trap day, sex, the interaction between trap day and sex, and time of day (*F*_4,40_ = 1.87, *P* = 0.135, *r*^2^ = 0.16, ΔAIC_c_ = 7.42) (Table 1), but the sex term did have a significant effect (*F*_1,40_ = 5.81, *P* = 0.021, *r*^2^ = 0.12), because females (least square mean 290.11 ± 1.47 mm) had smaller wing chord compared to males (least-square mean 295.43 ± 1.57 mm). Comparisons with a model including trap day, sex, and the interaction between trap day and sex (*F*_3,41_ = 2.50, *P* = 0.073, *r*^2^ = 0.16, ΔAIC_c_ = 4.88), a model including trap day and sex (*F*_2,41_ = 3.84, *P* = 0.030, *r*^2^ = 0.15, ΔAIC_c_ = 2.36) and a model including only sex (*F*_1,42_ = 7.79, *P* = 0.008, *r*^2^ = 0.15, ΔAIC_c_ = 0) indicated the parameters for the interaction term and time of day were uninformative (Table 1), and that the model with only the sex term was most parsimonious (Fig. 1C; Table 1). Statistical summaries for the models of body size metrics are shown in Table 1.

**Table 1:** Statistical summaries, including information criteria, for the linear model suites of Residual (body) mass, Keel depth, Wing chord, log-transformed heterophil/lymphocyte (Log H/L) ratio and log-transformed colony forming units of A1 and V1 bacteria in culture with plasma after 0, 2 and 4 hours (e.g. Log A1 at 0 h, Log A1 at 2 h)

**Model**	***n***	***k***	***I***	**D**	**S**	**S × D**	***T***_**c**_	***F***	***P***	***r*** ^**2**^	**ΔAIC**_**c**_	**LogL**
**Residual mass**												
D + S + S^*^D + T_c_	44	4	114.69 ± 37.47	−0.77 ± 0.22^*^	0.82 ± 1.90	−0.18 ± 0.22	0.25 ± 1.18^u^	3.23	0.022	0.25	6.75	−170.96
D + S + S^*^D	44	3	118.23 ± 33.10	−0.78 ± 0.21^*^	0.69 ± 1.78	−0.17 ± 0.21^u^		4.40	0.009	0.25	4.11	−170.86
D + S	44	2	114.97 ± 32.72	−0.76 ± 0.21^*^	0.68 ± 1.77^u^			6.33	0.004	0.24	2.27	−171.09
D	44	1	113.04 ± 31.99	−0.74 ± 0.21^*^				12.77	0.001	0.23	0	−171.05
Constant	44	0	−1.11 ± 1.95							0	9.37	−176.79
*Post hoc*												
D	49	1	111.59 ± 26.04	−0.73 ± 0.17^*^				18.43	<0.001	0.28		−188.00
**Keel depth**												
D + S + S^*^D + T_c_	44	4	−10.29 ± 5.46	0.12 ± 0.03^*^	−0.50 ± 0.26	0.03 ± 0.03	−0.20 ± 0.17^u^	4.64	0.004	0.32	2.95	−84.55
D + S + S^*^D	44	3	−13.51 ± 4.76	0.12 ± 0.03^*^	−0.41 ± 0.25	0.03 ± 0.03^u^		5.66	0.003	0.30	1.81	−85.19
D + S	44	2	−12.99 ± 4.70	0.12 ± 0.03^*^	−0.41 ± 0.25			8.21	0.001	0.29	0	−85.44
D	44	1	−11.79 ± 4.73	0.11 ± 0.03^*^				13.30	<0.001	0.24	0.29	−86.68
Constant	44	0	5.43 ± 0.29							0	10.09	−92.63
*Post hoc*												
D	49	1	111.59 ± 26.04	−0.73 ± 0.17^*^				2.23	<0.001	0.28		−94.29
**Wing chord**												
D + S + S^*^D + T_c_	45	4	293.48 ± 21.54	−0.02 ± 0.12	−2.66 ± 1.10	−0.02 ± 0.12	0.24 ± 0.70^u^	1.87	0.135	0.16	7.42	−151.13
D + S + S^*^D	45	3	297.07 ± 18.65	−0.03 ± 0.12	−2.78 ± 1.04	−0.01 ± 0.12^u^		2.50	0.073	0.16	4.88	−151.07
D + S	45	2	296.99 ± 18.42	−0.03 ± 0.12^u^	−2.78 ± 1.02			3.84	0.030	0.15	2.36	−150.96
S	45	1	292.76 ± 1.01		−2.810 ± 1.01			7.79	0.008	0.15	0	−150.88
Constant	45	0	292.58 ± 1.08							0	5.19	−154.52
*Post hoc*												
D	49	1	111.59 ± 26.04	−0.73 ± 0.17^*^				2.23	<0.001	0.28		−94.29
**Log H/L**												
D + S + S^*^D	42	3	3.67 ± 1.88	−0.03 ± 0.01^*^	0.17 ± 0.10	0.01 ± 0.01^u^		2.53	0.072	0.17	1.93	−40.34
D + S	42	2	3.64 ± 1.87	−0.03 ± 0.01^*^	0.17 ± 0.10			3.53	0.039	0.15	0	−40.54
D	42	1	3.40 ± 1.92	−0.02 ± 0.01				3.69	0.062	0.09	0.83	−42.06
S	42	1	−0.29 ± 0.10		0.16 ± 0.10			2.42	0.127	0.06	2.07	−42.68
Constant	42	0	−0.28 ± 0.10							0	2.21	−43.80

(Continued)

**Table 1:** Continued

**Model**	***n***	***k***	***I***	**D**	**S**	**S × D**	***T***_**c**_	***F***	***P***	***r*** ^**2**^	**ΔAIC**_**c**_	**LogL**
**Log A1 at 0 h**												
D + S + S^*^D	43	3	−4.33 ± 2.41	0.02 ± 0.02	−0.09 ± 0.13	0.01 ± 0.02^u^		0.81	0.495	0.06	4.72	−54.94
D + S	43	2	−4.38 ± 2.39	0.02 ± 0.02	−0.09 ± 0.13^u^			0.93	0.402	0.05	2.79	−55.13
D	43	1	−4.29 ± 2.37	0.02 ± 0.02^u^				1.44	0.237	0.03	0.83	−55.26
Constant	43	0	−1.45 ± 0.13							0	0	−55.89
**Log A1 at 2 h**												
D + S + S^*^D	43	3	−3.58 ± 2.14	0.03 ± 0.01^*^	−0.04 ± 0.12	−0.01 ± 0.01^u^		1.66	0.191	0.11	3.90	−49.88
D + S	43	2	−3.52 ± 2.14	0.03 ± 0.01	−0.04 ± 0.12^u^			2.05	0.143	0.09	2.33	−50.25
D	43	1	−3.49 ± 2.11	0.03 ± 0.01				4.08	0.050	0.09	0	−50.19
Constant	43	0	0.76 ± 0.12							0	1.76	−52.12
*Post hoc*												
D	56	1	−4.49 ± 2.10	0.03 ± 0.01^*^				5.96	0.018	0.10	0	−84.71
Constant	56	0	0.62 ± 0.15							0	3.63	−87.56
**Log A1 at 4 h**												
D + S + S^*^D	42	3	−1.80 ± 2.29	0.02 ± 0.01	0.04 ± 0.12	−0.01 ± 0.01^u^		0.96	0.423	0.07	4.30	−50.14
D + S	42	2	−1.63 ± 2.27	0.02 ± 0.01	0.04 ± 0.12^u^			1.16	0.325	0.06	2.35	−50.33
D	42	1	−1.71 ± 2.23	0.02 ± 0.01^u^				2.24	0.142	0.05	0.03	−50.28
Constant	42	0	1.63 ± 0.12							0	0	−51.31
*Post hoc*												
D	55	1	−6.76 ± 3.01	0.05 ± 0.02^*^				7.22	0.010	0.12	0	−101.82
Constant	55	0	1.32 ± 0.21							0	4.78	−105.25
**Log V1 at 0 h**												
D + S + S^*^D	43	3	−0.11 ± 1.75	0.00 ± 0.01	−0.16 ± 0.10	0.01 ± 0.01^u^		1.29	0.291	0.09	3.804	−41.165
D + S	43	2	−0.16 ± 1.75	0.00 ± 0.01	−0.16 ± 0.10			1.40	0.259	0.07	2.407	−41.624
D	43	1	0.00 ± 1.78	0.00 ± 0.01^u^				0.07	0.790	<0.01	2.798	−42.921
S	43	1	−0.45 ± 0.09		−0.16 ± 0.10			2.83	0.100	0.06	0	−41.522
Constant	43	0	−1.45 ± 0.13							0	0.558	−42.849

(Continued)

**Table 1:** Continued

**Model**	***n***	***k***	***I***	**D**	**S**	**S × D**	***T***_**c**_	***F***	***P***	***r*** ^**2**^	**ΔAIC**_**c**_	**LogL**
**Log V1 at 2 h**												
D + S + S^*^D	43	3	0.45 ± 1.18	0.01 ± 0.01	−0.08 ± 0.07	0.01 ± 0.01^u^		1.37	0.267	0.10	3.02	−24.41
D + S	43	2	0.42 ± 1.18	0.01 ± 0.01	−0.09 ± 0.07^u^			1.54	0.226	0.07	1.56	−24.84
D	43	1	0.51 ± 1.19	0.01 ± 0.01^u^				1.33	0.255	0.03	0.94	−25.63
Constant	43	0	1.88 ± 0.07							0	0	−26.21
**Log V1 at 4 h**												
D + S + S^*^D	40	3	1.68 ± 1.39	0.00 ± 0.01	0.03 ± 0.07	0.00 ± 0.01^u^		0.09	0.966	0.01	7.15	−24.16
D + S	40	2	1.66 ± 1.36	0.00 ± 0.01	0.02 ± 0.07^u^			0.13	0.879	0.01	4.54	−24.03
D	40	1	1.61 ± 2.23	0.00 ± 0.01^u^				0.13	0.716	<0.01	2.20	−23.97
Constant	40	0	2.11 ± 0.07							0	0	−23.92

Sample size (*n*), number of model parameters (*k*), *F* statistic (*F*), probability of the *F* statistic (P), coefficient of determination (*r*^2^), relative Akaike information criteria adjusted for small sample size (ΔAIC_c_), log-likelihood (LogL) and estimated parameters (±SE) for intercept (I) and fixed effects of trap day (D), sex (S), the interaction of trap day and sex (S × D) and time of capture (*T*_c_) in corresponding models are shown in the respective columns. Fixed effect abbreviations are also used to designate models (e.g. D + S + S × D indicates a model with fixed effects for trap day, sex and interaction of trap day and sex) and Constant indicates a model with no effects (i.e. the null model). *Post hoc* models constructed because a sex effect parameter was uninformative, are shown below the *Post hoc* label and have larger sample size because genetic sex was not ascertained for some individuals. Significant or uninformative fixed effect parameters are indicated by superscript * or u, respectively.

The heterophils and lymphocytes were the numerically dominant leukocytes present in blood smears, and the heterophil/lymphocyte ratio was not related to trap day. In 54 individuals for which we obtained differential counts, heterophils comprised 33.1 ± 14.3% (mean ± SD), lymphocytes 60.2 ± 16.0%, eosinophils 1.3 ± 1.7%, basophils 1.7 ± 1.6% and monocytes 3.8 ± 5.1% of the total leukocytes counted. In 42 individuals for which heterophil/lymphocyte ratio was available and sex was known, significant variation in log-transformed heterophil/lymphocyte ratio was not explained by trap day, sex and the interaction between sex and trap day (*F*_3,38_ = 2.53, *P* = 0.072, *r*^2^ = 0.17, ΔAIC_c_ = 1.93; Table 1). However, there was a trap-day effect (*F*_1,38_ = 4.45, *P* = 0.042, *r*^2^ = 0.10), though neither sex (*F*_1,38_ = 3.16, *P* = 0.084, *r*^2^ = 0.07) nor the interaction of sex and trap day (*F*_1,38_ = 0.60, *P* = 0.442, *r*^2^ = 0.01) was significant (Table 1). Comparisons with a model including trap day and sex (*F*_2,40_ = 3.53, *P* = 0.039, *r*^2^ = 0.15, ΔAIC_c_ = 0), a model including only trap day (*F*_1,41_ = 3.69, *P* = 0.062, *r*^2^ = 0.09, ΔAIC_c_ = 0.83) and a model including only sex (*F*_1,41_ = 2.42, *P* = 0.127, *r*^2^ = 0.06, ΔAIC_c_ = 2.07) indicated the parameter for the interaction term was uninformative and that the model with sex and trap-day effects was most parsimonious (but only explained 15% of the variance; Table 1). Log-transformed ratios were not correlated with residual body mass (ρ = 0.26, *P* = 0.095, *n* = 44) nor wing chord (ρ = −0.07, *P* = 0.651, *n* = 49). However, the log-transformed ratio of heterophils/lymphocytes was negatively correlated with keel depth (ρ = −0.32, *P* = 0.032, *n* = 44).

**Figure 2:**
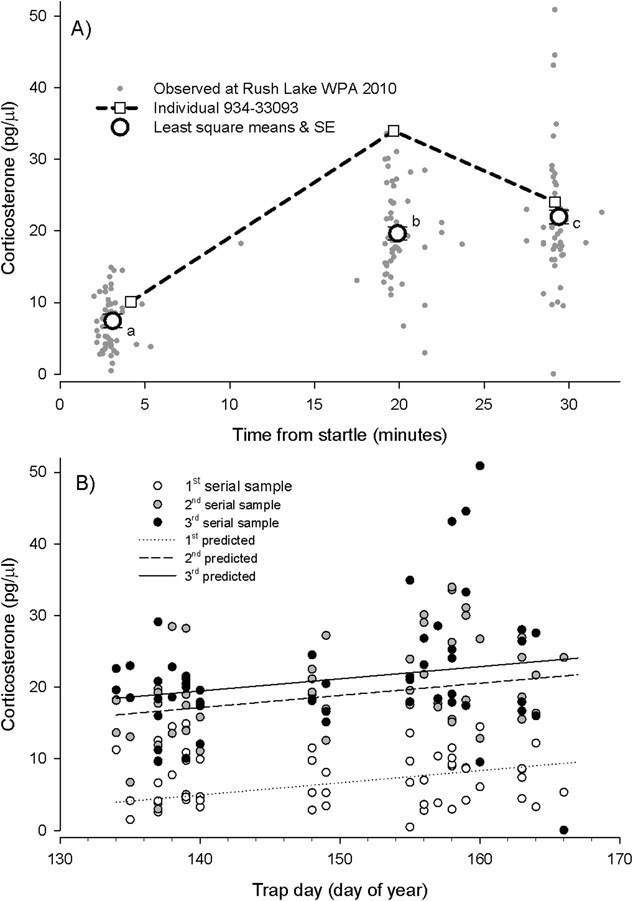
Plasma corticosterone concentration measured from nesting Franklin’s Gull adults increased significantly (**A**) with time from startle (*F*_2,108_ = 97.78, *P* < 0.001) and (**B**) with day of capture (i.e. trap day as ordinal day of year) (*F*_1,53_ = 7.05, *P* = 0.011) based on a linear mixed model with terms for trap day, serial sample number and adult id (random effect). In (A), grey dots indicate observed values, open circles indicate least square means (with SEs; different letters designate different means based on Tukey test) and the bold dashed line connecting open boxes represents concentrations from the same individual. In (B), open circles indicate observed first samples (with dotted line showing predicted), grey circles indicate second samples (with dashed line showing predicted) and filled circles indicate third samples (with the solid line showing predicted)

Corticosterone response was positively related to trap day, and the initial concentration was significantly lower than concentrations from the second and third serial plasma samples. Serially collected plasma samples exhibited corticosterone concentrations that increase from baseline concentrations to higher concentrations collected 20 minutes or more after the startle response. Maximum observed corticosterone concentrations occurred in either the second (20 individuals) or third (35 individuals) serial plasma sample for all 55 individuals for which three serial plasma samples were available. The mixed model with repeated measures with effects of trap day, sex, interaction between trap day and sex, serial sample number, time of day and adult ID (as a random effect) explained approximately 70% of the variation in corticosterone concentration, with approximately 25% of the variance associated with adult ID (the random effect), and significant (positive) effects of trap day (*F*_1,36_ = 4.80, *P* = 0.035) and sample number (*F*_2,80_ = 80.09, *P* < 0.001), and ΔAIC_c_ = 4.81 (Table 2). A Tukey test comparing the least-square means indicated the corticosterone concentrations for the first serial sample (7.28 ± 1.13 pg/μl) were significantly lower than those of the second (21.39 ± 1.13 pg/μl) and third samples (23.06 ± 1.13 pg/μl), but the second and third samples were not significantly different. Comparisons with a model including trap day, sex, the interaction between trap day and sex, serial sample number and adult ID (random effect) (*r*^2^ = 0.70, ΔAIC_c_ = 3.72), a model including trap day and sex (*F*_2,41_ = 6.32, *P* = 0.004, *r*^2^ = 0.24, ΔAIC_c_ = 2.27), a model including trap day, sex, serial sample number and adult ID (random effect) (*r*^2^ = 0.70, ΔAIC_c_ = 0.40), a model including trap day, serial sample number and adult ID (random effect) (*r*^2^ = 0.70, ΔAIC_c_ = 0.93), and a model including serial sample number and adult ID (random effect) (*r*^2^ = 0.70, ΔAIC_c_ = 0) indicated parameters for time of capture, sex and interaction between trap day and sex were uninformative (Table 2). A *post hoc* mixed model with repeated measures including trap day, serial sample number and adult ID (random effect) (for a sample size of 55 individuals) explained 70% of the variation in corticosterone concentration, with approximately 29% of the variance associated with adult (the random effect) and significant (positive) effects of trap day (*F*_1,53_ = 7.05, *P* = 0.011) and sample number (*F*_2,108_ = 97.78, *P* < 0.001) (Fig. 2; Table 2). A Tukey test comparing the least square means from this model indicated the corticosterone concentrations for the first serial sample (7.44 ± 0.94 pg/μl) were significantly lower than those of the second (19.65 ± 0.94 pg/μl), which were significantly lower than those of the third samples (21.97 ± 0.94 pg/μl) (Fig. 2). However, an alternative *post hoc* mixed model with repeated measures including only serial sample number and adult ID (random effect) also explained 70% of the variance and had similar parsimony (ΔAIC_c_ = 0.99; Table 2). The observed elevation in plasma corticosterone (Fig. 2A) is consistent with hormonal response to exposure to an acute stress in birds (Siegel, 1980). None of the serial plasma corticosterone concentrations were correlated with residual body mass, keel depth or wing chord (−0.18 < ρ < 0.19, *P* > 0.184, *n* = 45 for all comparisons) nor were they correlated with log-transformed ratio of heterophils/lymphocytes (−0.22 < ρ < −0.01, *P* > 0.118, *n* ≥ 52 for all comparisons).

**Table 2:** Statistical summaries, including information criteria, for the repeated measures, mixed model suite for corticosterone concentration (Cort, in pg/μl)

**Cort**	***n***	***k***	***I***	**D**	**S**	**S × D**	***T***_**c**_	**Second**	**Third**	***r*** ^**2**^	**ΔAIC**_**c**_	**LogL**
D + S + S^*^D+ T_c_ + N	123	6	−20.74 ± 16.80	0.21 ± 0.10^*^	−1.21 ± 0.86	−0.12 ± 0.10	−0.43 ± 0.53^u^	14.11 ± 1.37^*^	15.78 ± 1.37	0.71	4.81	−412.43
D + S + S^*^D+ N	123	5	−27.06 ± 14.82	0.22 ± 0.10^*^	−0.97 ± 0.81	−0.13 ± 0.10^u^		14.11 ± 1.37^*^	15.78 ± 1.37	0.70	3.72	−412.99
D + S + N	123	4	−25.20 ± 14.91	0.21 ± 0.10^*^	−0.95 ± 0.81^u^			14.11 ± 1.37^*^	15.78 ± 1.37	0.70	0.40	−412.42
D + N	123	3	−23.58 ± 14.92	0.20 ± 0.10^*^				14.11 ± 1.37^*^	15.78 ± 1.37	0.70	0.93	−413.75
N	123	2	7.03 ± 1.15					14.11 ± 1.37^*^	15.78 ± 1.37	0.70	0	−414.33
*Post hoc*												
D + N	165	3	−18.86 ± 9.95	0.18 ± 0.07^*^				12.21 ± 1.12^*^	14.54 ± 1.12^*^	0.70	0	−548.24
N	165	2	7.44 ± 0.96					12.21 ± 1.12^*^	14.54 ± 1.12^*^	0	0.99	−549.77

Sample size (*n*), number of model parameters (*k*), coefficient of determination (*r*^2^), relative Akaike information criteria adjusted for small sample size (ΔAIC_c_), log-likelihood (LogL) and estimated parameters (±SE) for intercept (I) and fixed effects of trap day (D), sex (S), the interaction of trap day and sex (S*D), Time of capture (*T*_c_) and serial sample (second or third) in corresponding models are shown in the respective columns. Fixed effect abbreviations are also used to designate models (e.g. D + S + S × D indicates a model with fixed effects for trap day, sex and interaction of trap day and sex), but *N* denotes sample number effect. *Post hoc* models shown below the *Post hoc* label have larger sample size because genetic sex was not ascertained for some individuals. Significant or uninformative fixed effect parameters are indicated by superscript * or u, respectively.

**Figure 3:**
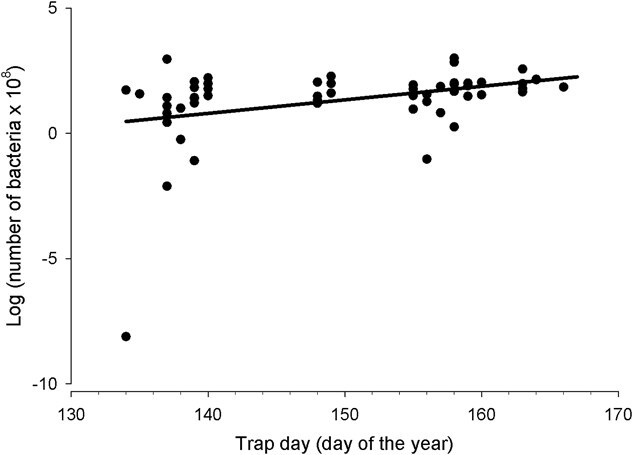
Results of a bactericidal assay of plasma collected from incubating Franklin’s Gull adults indicated the number of bacteria in the A1 (complement-sensitive) bacteria culture after four hours incubation was positively related to trap day (*F*_1,54_ = 7.22, *P* = 0.010, *r*^2^ = 0.12). Filled circles indicate observed values, and the solid lines represent the linear regression fit to the data

Population size of the A1 (complement-sensitive) bacteria culture after 2- and 4-hour incubation time increased with trap day. For 43 observations of known-sex individuals, variation in log-transformed estimates of the number of A1 bacteria at 2 hours was not explained by the trap day, sex and trap-day–sex-interaction model (*F*_3,39_ = 1.66, *P* = 0.191, *r*^2^ = 0.11, ΔAIC_c_ = 3.90), with sex (*F*_1,39_ = 0.11, *P* = 0.742, *r*^2^ < 0.01) and sex–trap day interaction effects not significant (*F*_1,39_ = 0.91, *P* = 0.346, *r*^2^ = 0.02), but the trap-day effect was significant (*F*_1,39_ = 4.16, *P* = 0.048, *r*^2^ = 0.11) (Table 1). Comparisons with a model including trap day and sex (*F*_2,40_ = 2.05, *P* = 0.143, *r*^2^ = 0.09, ΔAIC_c_ = 2.33) and a model including only trap day (*F*_1,41_ = 4.07, *P* = 0.050, *r*^2^ = 0.09, ΔAIC_c_ = 0) indicated the parameters for the sex and interaction terms were uninformative (Table 1). A *post hoc* model of log-transformed estimates of the number of bacteria in the A1(complement-sensitive) culture at 2-hours (*F*_1,54_ = 5.96, *P* = 0.018, *r*^2^ = 0.10, ΔAIC_c_ = 0; Fig. 3) with a (positive) trap-day effect was more parsimonious than a model without the trap-day effect (ΔAIC_c_ = 3.627) (Table 1). The trap day, sex, trap day and sex interaction model did not explain variation in the log-transformed number of A1 bacteria at 4 hours for 42 observations of known-sex individuals (*F*_3,38_ = 0.958, *P* = 0.423, *r*^2^ = 0.07, ΔAIC_c_ = 4.30) and none of the effects (trap day, sex, interaction between sex and trap day) were significant (Table 1). Comparisons with a model including trap day and sex (*F*_2,39_ = 1.16, *P* = 0.325, *r*^2^ = 0.06, ΔAIC_c_ = 2.35) and a model including only trap day (*F*_1,40_ = 2.24, *P* = 0.142, *r*^2^ = 0.05, ΔAIC_c_ = 0.03) indicated none of the parameters were informative (Table 1). However, a *post hoc* model of log-transformed estimates of the number of bacteria in the A1(complement-sensitive) culture at 4 hours (*F*_1,53_ = 7.22, *P* = 0.001, *r*^2^ = 0.12, ΔAIC_c_ = 0; Fig. 3) increased significantly with trap day (Table 1) and was more parsimonious than a model without the trap-day effect (ΔAIC_c_ = 3.627) (Table 1). Log-transformed estimates of the initial number of bacteria in the A1 culture did not differ with trap day, sex or the interaction of sex and trap day in any models in the suite (Table 1). Similarly, for individuals for which sex was known and V1 (complement-resistant) bacteria counts were available, log-transformed number of V1 bacteria at initiation (*F*_3,39_ = 1.29, *P* = 0.291, *r*^2^ = 0.09), 2 hours (*F*_3,39_ = 1.37, *P* = 0.267, *r*^2^ = 0.10) and 4 hours (*F*_3,39_ = 0.09, *P* = 0.967, *r*^2^ = 0.01) incubation were not significantly explained by trap day, sex and the interaction between sex and trap day (with none of the terms having significant effects) nor were any of these effects informative or part of the most parsimonious models in candidate suites (Table 1). Log-transformed number of bacteria in the V1 culture at zero-hour incubation was positively correlated with log-transformed heterophil/lymphocyte ratio (ρ = 0.34, *P* = 0.019, *n* = 48) and log-transformed number of bacteria in the V1 culture at 2-hour incubation was negatively correlated with residual body mass (ρ = −0.31, *P* = 0.038, *n* = 45), but none of the other estimates of bacteria population size were significantly correlated with residual body mass, keel depth, wing chord and heterophil/lymphocyte ratio nor with serial corticosterone concentration.

## Discussion

Understanding patterns of seasonal variation in the condition of breeding adults is critical to predicting how factors affecting the timing of nesting in migratory birds will impact reproductive success. Structural size of Franklin’s Gull chicks at hatching varies seasonally, due to both photoperiod (an environmental signal) effects and maternal effects (Clark and Reed, 2012). Environmental cues vary seasonally and can produce changes in adult physiology; however, no information is available on how adult condition and physiology change during the breeding season in Franklin’s Gull nor from many other migratory species.

Our observation that residual body mass (i.e. body mass deviation from skeletal size) of nesting adult Franklin’s Gulls was smaller in individuals that initiated nesting later in the breeding season (Fig. 1) is consistent with reports for other species, but the causal mechanisms behind this pattern are unknown. Kitaysky *et al.* (1999) found that body condition decreased in late-nesting Kittiwakes and conjectured that local ecological factors (e.g. declining food availability) were the proximate mechanisms responsible for the pattern. Other studies have reported similar trends in body mass or body condition declining in adults with later-initiated nests (Thogmartin and Johnson, 1999; Devries *et al.*, 2008; Warren *et al.*, 2014). But hypotheses for mechanisms driving these relationships include environmental conditions experienced prior to the breeding season (Thogmartin and Johnson, 1999) or upon arrival at the breeding site (Devries *et al.*, 2008). If later nesting birds arrive at breeding sites later because they are in poorer condition and took longer to complete migration, a similar pattern in body condition and timing of nesting might be observed. Both *et al.* (2005) showed that both arrival and breeding dates in Pied Flycatchers (*Ficedula hypoleuca*) were dependent on temperatures on their main staging grounds and Rockwell *et al.* (2012) found that winter temperatures carried over to affect arrival and reproductive success in Kirtland’s Warbler (*Setophaga kirtlandi*). Golet and Irons (1999) suggest that breeding adult Kittiwakes (*Rissa tridactyla*) compromise their body condition because they are investing resources in chicks. We captured nesting Franklin’s Gull adults within the first week of continuous incubation (and prior to the onset of food provisioning of offspring), yet still observed lower residual body mass in individuals nesting later in the season. Information on the temporal dynamics of macroinvertebrates associated with the Northern Plains is unavailable but could provide data to determine if local resource limitation is correlated with body metrics in Franklin’s gull during the incubation period.

The finding that keel depth increased across the breeding season could be explained by trade-offs between adult and chick condition, or factors related to seasonal condition prior to breeding. Similar to our findings, Manuel Neto and Gosler (2010) found that breast muscle size and protein reserves decreased across the breeding season in Savi’s Warblers (*Locustella luscinioides*), which is thought to influence future survival. This could be due to breeding adults compromising their own body condition to offset diminished resources for chicks associated with late-season nests, or due to factors (e.g. inclement weather delaying migration) experienced prior to arrival as explained for size metrics in the previous paragraph. Carry-over effects from the non-breeding season better explained nesting phenology in several long-distance migratory birds in Europe than conditions at the breeding areas (Finch *et al.*, 2014), and Cooper *et al.* (2015) experimentally demonstrated that winter conditions delay arrival dates in the American Redstart (*Setophaga ruticilla*).

We did not find evidence of seasonal variation in the heterophil/lymphocyte ratios during the nest initiation period in Franklin’s Gull, which is inconsistent with carry-over effects related to nesting phenology. Heterophils are components of the innate immune system, and lymphocytes are primary components of the vertebrate acquired immune system (Bone and Moore, 2008). An increase in the number of heterophils relative to lymphocytes can indicate long-term changes in physiology or an increase in vulnerability to infection (Gross and Siegel, 1983; Vleck *et al.*, 2000). Several studies indicate that incubating adults exhibit an increase in heterophil/lymphocyte ratios compared to non-incubating adults during the breeding season (Ots and Hõrak, 1996; Hõrak *et al.*, 1998; Hanssen *et al.*, 2005) unlike our observations.

Later nesting Franklin’s Gull adults are potentially more sensitive to a perceived immediate threat (i.e. stressor) than are earlier nesting adults. Birds that initiate nesting later in the season show an elevated corticosterone response when exposed to an acute stressor compared to birds that initiate nesting earlier in the season (Fig. 2). Adams *et al.* (2005) found that the corticosterone response increases in birds during late incubation, which is similar to findings that variation in corticosterone levels during the breeding season are related to reproductive stage of the adult (Pereyra and Wingfield, 2003), but we attempted to control for these effects by capturing all individuals in the early stage of incubation. Other findings indicate that both prolactin (the concentration of which generally increases during incubation) and corticosterone interact synergistically during nesting (Angelier *et al.*, 2013). Edwards *et al.* (2013) found that variation in baseline corticosterone concentrations was established prior to incubation in two species of shorebird. Our observations indicate there is seasonal variation in sensitivity to an immediate stressor not related to differences in the reproductive cycle of adult Franklin’s Gull.

Suppression of bacterial growth by plasma complement proteins was evident in early-nesting gulls compared to late-nesting gulls (Fig. 3) and could be due to reproductive effort or the nest-site environment. Constitutive immunity in vertebrates can be assessed by the bacterial capacity of plasma (Millet *et al.*, 2007). Tree Swallow (*Tachycineta bicolor*) females that initiate nesting early also have plasma with a stronger ability to kill bacteria than later nesting females (Ardia, 2005). But experimentally increasing the reproductive effort resulted in a decreased bacteria-killing ability (Ardia, 2005), which supports the hypothesis that there is a trade-off between reproductive investment and adult survival in addition to within-season factors affecting immune function. However, Møller *et al.* (2003) found that birds experience seasonal changes in the impact of parasites, with additional variation in immune function related to nest type and location. If nest-site characteristics in Franklin’s Gull vary within the season (e.g. later nesting individuals have more limited sites or higher density sites than early-nesting individuals), this could also explain the patterns we observed.

Within-season variation in condition may be related to time constraints through resource allocation for self-maintenance and offspring. Many researchers have conjectured that post-migratory residual body stores are critical for successful breeding (Ankney and Macinnes, 1978; Drent and Daan, 1980; Ebbinge and Spaans, 1995; Ryder, 1970; Sandberg and Moore, 1996). Perrins (1970) hypothesized that breeding birds laying eggs later in the season experienced a shortage of food during egg formation, resulting in offspring that are unable to fully profit from the seasonal peak in food availability seen in most temperate ecosystems. The relative importance of each factor (residual fat stores versus local food resources) has been more broadly expanded to contrast species in which a capital (e.g. fat stores) versus income (e.g. local food resources) breeding strategy is evident in the reproductive life history (Drent and Daan, 1980). When the reproductive value of offspring varies within season, capital breeding is favoured (Ejsmond *et al.*, 2015). Reproductive value (e.g. structural size at hatching, post-hatching survival) of Franklin’s Gull chicks decline as the breeding season progresses (Berg, 2009; Clark and Reed, 2012), which theoretically predicts capital breeding. However, at present, not enough information is available to determine if Franklin’s Gull is a capital breeder.

Regardless of breeding strategy, the timing of nesting has significant fitness consequences for offspring in this species (Berg, 2009; Clark and Reed, 2012). We observed within-season differences in multiple metrics of adult size and physiology during the first part of incubation (and therefore before costs of parental care in chick provisioning are incurred). However, it is not known if these seasonal differences are present during the latter stages (e.g. the rapid yolk development stage) of egg development. Furthermore, our observations of less flight muscle and increased corticosterone response with later nesting were similar for both females and males, suggesting that changes in condition at the onset of breeding are not restricted to costs incurred by egg formation alone. Little is known about physiological condition before and during migration, how food resources vary seasonally, how egg constituents vary seasonally and how all of these relate to the timing of nesting in Franklin’s Gull. Our results indicate that late-nesting adults have smaller relative mass and less pectoral muscle compared to early-nesting adults, which likely compounds differences in survival of late-season versus early-season chicks. Determining whether resources acquired outside the breeding area (i.e. capital) or locally (i.e. income) underlie the seasonal patterns in adult condition and chick characteristics is critical to understanding the ecology of Franklin’s Gull and other long-lived birds nesting in the Northern Plains.

Finally, within-nesting-season variation in adult physiology potentially interacts with effects of climate change. Advanced timing of nesting corresponding with climatic warming has been documented in many birds (Bates *et al.*, 2023). Seasonal changes in timing can compromise population dynamics and viability if the shifts create a mismatch (Miller-Rushing *et al.*, 2010), for instance, between offspring production and peak food availability (Visser *et al.*, 1998; Reed *et al.*, 2013). If the timing of Franklin’s Gull nesting advances with warming climate, we expect the corticosterone response of adults to shift, potentially mismatching the stress response and persistence of nesting adults with the reproductive value of their current versus future offspring. Such physiological mechanisms can be incorporated into models to better predict population response to climate change (McLean *et al.*, 2016), but further studies are needed to quantify effects of the mechanisms.

## Data Availability

The data underlying this article will be shared on reasonable request to the corresponding author.
